# Pathological upgrading and upstaging at radical prostatectomy in Jamaican men with low-risk prostate cancer

**DOI:** 10.3332/ecancer.2019.971

**Published:** 2019-10-29

**Authors:** Belinda F Morrison, William D Aiken, Gareth Reid, Richard Mayhew, Barrie Hanchard

**Affiliations:** University of the West Indies, Mona PO, Kingston 7, Jamaica

**Keywords:** low-risk prostate cancer, Jamaica, upgrading, upstaging, active surveillance

## Abstract

Several studies suggest race-based health disparities in men with low-risk prostate cancer (PCa), with African American males having poorer oncological outcomes. We sought to determine the prevalence and predictors of pathological upgrading and upstaging in Jamaican men with low-risk PCa treated with radical prostatectomy (RP). Data on 141 men who met the National Comprehensive Cancer Network criteria for low-risk PCa and underwent RP at a single institution were reviewed. All men had a transrectal ultrasound-guided biopsy. Pre-operative clinical and final pathological data were obtained. Data were summarised as means and standard deviations or percentages as appropriate. Bivariate analyses such as independent samples t-tests and chi-square tables were conducted and logistic regression models were estimated to predict upgrading (>Gleason 6) and upstaging (p ≥ T3).

The mean age was 59.5 ± 7.8 years with mean prostate specific antigen (PSA) of 6.6 ± 2 ng/mL. A total of 48.3% of men were upgraded and 11.4% were upstaged. Bivariate analyses indicated that PSA (p = 0.008) and percentage positive cores (p = 0.002) were associated with upgrading. PSA (p = 0.042) and percentage positive cores (p = 0.003) were significantly associated with upstaging. The odds of upgrading increased with increased PSA levels (OR 1.40, 95% CI 1.05–1.87, p = 0.021) or increased percentage positive cores (OR 8.27, 95% CI 2.19–31.16, p = 0.002). The odds of upstaging increased with increased PSA levels (OR 1.4, 95% CI 1.01–1.96, p = 0.046) and with increased percentages positive cores (OR 11.4; 95% CI 2.06–63.09, p = 0.005). Jamaican men with low-risk PCa are at high risk of pathological upgrading and upstaging at RP. These findings should be taken into consideration when discussing treatment options with these patients.

## Introduction

Jamaican men, like most black males, are at high risk of developing prostate cancer (PCa) [1]. At least 90% of Jamaicans are of African ethnicity, a feature that appears to confer an increased biological aggressiveness of PCa [2]. PCa has the highest incidence of cancers in the island and accounts for the highest mortality of all cancers in Jamaica [1, 3]. Most men diagnosed with PCa in the United States have low-risk disease due to a prior downward stage migration attributed to widespread screening [4]. However, in Jamaica where screening is not widespread, several reports suggest that diagnosis tends to be with locally advanced or metastatic disease [5–7]. Hospital databases in Jamaica report that low-risk PCa is seen in approximately 10% of all cases of PCa. Consequently, curative treatment with radical prostatectomy (RP) or observation through active surveillance is offered far less frequently than hormonal manipulation with androgen deprivation therapy [8].

There is great incongruity between prostate biopsy grade and clinical stage and RP grade and pathological stage. Estimates suggest that over 50% of patients classified with low-risk PCa are either upgraded or upstaged at RP [9, 10]. There are divergent results from studies evaluating the association of black race with increased risk of pathological upgrading or upstaging at RP [9, 11–13]. Results of some of these studies pose valid concerns regarding the suitability of offering active surveillance as a therapeutic option to black males with apparent low-risk PCa.

We therefore sought to determine the prevalence of pathological upgrading and upstaging in Jamaican men with low-risk PCa treated with RP between 2000 and 2015. Additionally, we sought to determine the clinicopathological variables which increased the risk of upgrading or upstaging.

## Methods

### Study Design

A retrospective analysis of the Pathology database at the University Hospital of the West Indies, Kingston, Jamaica was conducted. The University Hospital of the West Indies is a large tertiary facility in Kingston Jamaica and is one of the three public facilities providing full-time urological care for patients in Jamaica. All men diagnosed with histologically confirmed low-risk PCa, who underwent radical retropubic prostatectomies at the institution between 1 January 2000 and 31 December 2015 were identified. All men treated between 2004 and 2015 had systematic transrectal ultrasound-guided biopsies with a minimum of 12 cores taken (Sextant biopsies were performed prior to 2004). Biopsy and RP specimens were reviewed by pathologists at the University Hospital of the West Indies. RPs were performed in an open retropubic fashion by several urologists at the University Hospital of the West Indies, Kingston, Jamaica. Patients who received neoadjuvant therapy were excluded.

Low-risk PCa was defined using the National Comprehensive Cancer Network (NCCN) (2010) criteria as persons with a life expectancy of at least 10 years, PSA < 10 ng/mL at diagnosis, clinical T stage of PCa of T1c or T2a and Gleason score of ≤ 6 [14].

Pre-operative clinical and pathological variables included patient age, clinical stage, PSA, Gleason score and percentage positive cores on biopsy (overall percentage of total cores with cancer) and number of positive cores. Surgical pathology of the RP specimen included pathological stage, Gleason score and presence of adverse pathological features (seminal vesicle invasion, extracapsular extension and positive surgical margins). Upgrading was defined as an increase in Gleason score between the biopsy and the RP sample. Upstaging was defined as pathological stage T3 and greater.

### Statistical analysis

Data were summarised as means and standard deviations or percentages as appropriate. Bivariate analyses including independent samples *t*-tests, chi-squared tests or Fisher’s exact tests were conducted to determine which characteristics were associated with upgrading and upstaging. After this, two separate logistic regression models were estimated predicting upgrading and upstaging, respectively. These models were estimated in order to quantify the bivariate associations that were found.

## Results

### Baseline patient characteristics

The cohort consisted of 141 men. The mean patient age was 59.5 ± 7.8 years (range: 37–75). The median PSA was 6.7 ng/mL (range: 0.2–9.9 ng/mL). Most patients (82.3%) had clinical stage T1c disease while 17.7% had clinical stage T2a disease. The median number of positive cores on prostate biopsies was two (range: 1–6). Of the patients in the cohort, 60.7% had less than 10% positive cores while 21.4% had greater than 20% involvement (Table 1).

### Prevalence of upgrading and upstaging

Of the patients in the cohort, 48.3% (57 of 118) were upgraded (There were missing data on grade in the remaining 23 patients) (Table 1). Gleason 7 disease was found in 98% (56 of 57 patients) of these and Gleason 8 (1 of 57 patients) in the remainder. Patients with Gleason 7 adenocarcinoma were further classified as Gleason 3+4 (International Society of Urological Pathologists (ISUP) grade group II) (34 of 56 patients), Gleason 4+3 (ISUP grade group III) (11 of 56 patients) and no sub-classification was provided in 11 patients. Additionally, 11.4% of patients (16 of 140 patients) were upstaged and the positive margin rate was 23.5% (There were missing data on stage in one patient) (Table 2). Of the patients upstaged, 18.8% (3) were T3aN0/1, 56.3% (9) were T3bN0/1 and 43.8% (7) were N1 disease.

### Predictors of upgrading and upstaging

Tests of association indicated that median PSA levels were higher among men who experienced upgrading compared to those who did not (median = 7.0 versus 6.0 ng/mL; *p* = 0.014) (Table 2). Chi-square tests of association indicated that the proportion of men who experienced upgrading varied significantly with the percentage of positive cores (*p* = 0.002) (Table 2). There was no association between upgrading and age, number of positive cores or clinical stage (Table 2). A logistic regression model predicting pathological upgrading indicated that, for each unit increase of PSA level, the odds of upgrading increased by 40% (OR = 1.40, 95% CI 1.05–1.87, *p* = 0.021). The odds of upgrading were eight times higher among patients with greater than 20% positive cores when compared to patients with less than 20% positive cores (OR = 8.27, 95% CI 2.19–31.16, *p* = 0.002).

Tests of association indicated that median PSA levels were higher among men who experienced upstaging compared to those who did not (median = 7.8 versus 6.4; *p* = 0.042) (Table 3). Chi-square tests of association indicated that the proportion of men who experienced upstaging varied significantly with the percentage of positive cores (*p* = 0.003). There was no association between upstaging and age, number of positive cores or clinical stage (Table 3). A crude logistic regression model predicting pathological upstaging indicated that the odds of upstaging was increased by 40% (OR 1.4, 95% CI 1.01–1.96, *p* = 0.046) for each unit increase in PSA level. Another crude logistic regression model predicting pathological upstaging indicated that patients with greater than 20% positive cores had more than 10 times the odds of experiencing upstaging compared to those with less than 10% (OR 11.5, 95% CI 2.13–61.84, *p* = 0.004). In a model including both PSA levels and percentage cancer cells, only greater than 20% positive cores continued to predict upstage status (OR 11.4, 95% CI 2.06–63.09, *p* = 0.005).

## Discussion

In a cohort of Jamaican men with low-risk PCa (PSA < 10 ng/mL, cT1c or cT2a and Gleason score of ≤ 6) treated with radical retropubic prostatectomy, it was found that approximately 60% of these men had either pathological upgrading or upstaging. Of these men, 48.3% were upgraded to Gleason 7 and 8 and 11.4% were upstaged to T3/N0-1 disease. Predictors of upgrading and upstaging were higher PSA and higher percentage positive cores.

Previously published prevalence rates of pathological upgrading and upstaging in low-risk PCa are similarly high as in our study. In a cohort of over 10,000 men in the Surveillance Epidemiology and End Results Program (SEER) database, 44% of cases were upgraded to Gleason 7–10 and 9.7% were upstaged [10]. Similarly, pathological upgrading and upstaging were found in 43.3% and 9%, respectively, of the over 48,000 men with low-risk PCa evaluated between 2010 and 2013 [9]. In an active surveillance cohort from Guadeloupe, a Caribbean island with a similar ethnic population to Jamaica, upgrading was reported in 52% of the 73 men who eventually underwent RP [15].

Our results are similar to other studies that have shown associations between PSA levels as well as percentage positive cores and upgrading and upstaging [9, 10, 12, 16, 17]. Dinh *et al* [10] reported that a PSA greater than 5.0 ng/mL was associated with upgrading and upstaging in men with low-risk PCa (*p* < 0.01). Dinh *et al* [10] reported that positive cores greater than 25% were associated with upgrading and upstaging (*p* < 0.001). Unlike other studies, we did not find an association between patient age and pathological upgrading and upstaging. Age greater than 60 years has been found in previous studies to be associated with pathological upgrading and upstaging [10]. Other useful predictors of upgrading and upstaging not evaluated in our study include prostate volume and obesity [16–18].

Several studies have reported that the black race is a significant predictor of pathological upgrading and upstaging in men with low-risk PCa [9, 11, 12]. In the seminal paper by Sundi *et al* [11], 256 African American (AA) men and 1,473 Caucasian American (CA) men with very low-risk PCa were compared to determine the oncological outcomes after RP. The study reported that AA men had significantly higher rates of pathological upgrading (27.3% versus 14.4%, *p* < 0.01) and adverse pathological features (14.1% versus 7.7%, *p* < 0.01) after surgery compared to CA men. A major limitation of the study was the small number of AA men included in the cohort. In addition, this study included only men with very low-risk PCa. Maurice *et al* [9] have reported on the largest cohort of AA patients with low-risk PCa using data from the US National Cancer Database. A total of 5,411 AA men were compared to 43,062 non-AA men with low-risk PCa. In this study, the AA race was a significant predictor of pathological upgrading and upstaging (OR 1.2, 95% CI 1.1–1.3, *p* < 0.01). Despite these findings, other studies have not found a significant association between black race and pathological upgrading or upstaging [13, 19–21]. A major limitation of these studies is underrepresentation of AA men with the largest study including 355 AA men.

Upgrading and upstaging may be due to a number of factors. These include under-sampling at the time of biopsy, atypical location of cancers or variability in pathologist’s expertise. Transition from sextant biopsies to increasing the minimum number of cores to 12 on systematic prostate biopsies has reduced rates of under-sampling [22]. However, the atypical location of cancers may still result in under-sampling. Sundi *et al* [23] reported that AA males are more likely to have a unique location of cancers in the anterior zone of the prostate, which is not typically included in systematic biopsy regimes. In men with very low-risk PCa, Sundi *et al* [23] found that AA men were more likely than CA men to have an anterior dominant nodule (50.6% versus 28.7%, *p* = 0.003) and in those patients who were upgraded, AA men were more likely to have an anterior nodule (59.4% versus 0%, *p* = 0.001). Tiguert *et al* [24] also reported that AA race was a predictor of anterior zone tumours, which was associated with a higher rate of positive surgical margins. Pathologists’ expertise is critical in accurately classifying patients with PCa. Majoros *et al* [25] reported that pathologists in non-academic low-volume centres, where less than 100 pathology specimens were reviewed annually, were more likely to have discordance in results compared to pathologists from high volume centres. We consider our large-volume academic pathology department as a strength to our study where fairly high concordance has been previously reported [26].

Our rate of positive surgical margins was 23.5%. This rate of positive surgical margins could be due to the biology of PCa in our patients or surgical technical challenges [27, 28]. Previous reports have suggested more technically challenging surgery in the black male due to the smaller mid-pelvic area and steeper symphysis angle [29]. This consequently results in the black race being a predictor for positive surgical margins, particularly at the apex [28, 30].

The American Urological Association (AUA) guideline on management of clinically localised PCa suggests that men with low-risk PCa may be offered active surveillance as a management option, regardless of race [31]. Since there is compelling evidence that the black race significantly increases the risk of adverse pathological features in men with biopsies demonstrating low-risk disease, eligible black patients for active surveillance must be counselled about these potential risks. It is presumed that adverse pathological features are associated with worse clinical outcomes in these patients. Robust retrospective data from the SEER database comparing mortality in low-risk AA (7,532) and CA males (43,792) who would be eligible for active surveillance showed that AA males had significantly higher disease-specific mortality compared to CA males (adjusted hazard ratio (AHR) –1.45, *p* = 0.032) [32]. Conversely, Leapman *et al* [13] using data from the SEARCH database found that there was no difference in 5-year recurrence-free survival rates between 355 AA and 540 CA men with low-risk PCa who were eligible for active surveillance (73.4% versus 78.4%, *p* = 0.18). Despite these differing results, there are no data documenting reduced overall or disease-specific survival of AA men compared to CA men enrolled in active surveillance cohorts. Iremashvili *et al* [33] reported that AA men who are part of an active surveillance cohort at the University of Miami were more likely to have progression on re-biopsy than CA men during surveillance (HR 3.87–4.12). Similarly, Abern *et al* [34] reported that AA men on an active surveillance cohort at Duke Prostate Centre were more likely to discontinue surveillance and choose treatment than CA males (HR 2.93, *p* = 0.01). AA men in the active surveillance cohort from Johns Hopkins were more likely to have reclassification to Gleason 7 or greater, compared to CA men, leading to discontinuation of surveillance (36% versus 16%, *p* < 0.001) [35]. All three studies were limited by very small sample sizes of black men and did not report on long-term clinical outcomes. In a much larger active surveillance cohort in Guadeloupe, comprised of 234 Afro-Caribbean men with mostly low-risk and a few favourable intermediate-risk PCas, good clinical outcomes were reported [15]. At a median follow-up of 4 years, only 58.1% of men remained under surveillance. Of those treated (35%), the most common indication was tumour volume increase or upgrading. However, there was no cancer-specific death and overall survival at 5 and 10 years was 98.5% and 90%, respectively. It appeared that despite the high rates of pathological upgrading in the cohort (52%), active surveillance was associated with good clinical outcome.

We acknowledge limitations to our study. This was a retrospective, single-centre series from a tertiary academic institution where RPs were performed by several urologists. Pathological reporting was not done by a central uro-pathologist, possibly introducing detection bias. Patients included in the analysis between 2000 and 2003 (30 patients) had sextant biopsies done, also introducing detection bias. We were not able to provide specific details on biopsy characteristics such as single core tumour volume, prostate volume, PSA density or tumour location. Our study only included patients with low-risk PCa who had surgery and did not report on outcomes in all low-risk PCa patients. No assessment of the long-term oncologic outcome was done in our patients.

We consider our study meaningful as it adds to the body of knowledge on PCa in Afro-Caribbean men. Prostate cancer is not only very common in many Caribbean nations but it is also the leading cause of male cancer deaths [36]. The Caribbean region has the unenviable designation as having the highest mortality rate from PCa worldwide [37]. Though there are shared genetic determinants for PCa, between AA and Afro-Caribbean men, it is worthwhile evaluating differences in pathology, clinical presentation and outcomes in black men in the Caribbean. Our study revealed a high rate of pathological upgrading and upstaging in Jamaican men with low-risk PCa. We believe that active surveillance may be offered to these men; however, they must be counselled about the high risk of adverse pathological features. We believe that risk stratification of these patients utilising tools such as multiparametric magnetic resonance imaging, which became available in Jamaica in 2017; genomic testing and specific selection criteria may contribute to accurately classifying patients and ensuring good long-term clinical outcomes [10, 38–40]. In addition, perhaps amended biopsy protocols including cores from the anterior zone of the prostate may be suggested in AA and Afro-Caribbean men. Further prospective studies evaluating outcomes of low-risk men with PCa enrolled in active surveillance cohorts in Jamaica are needed.

## Conclusion

Jamaican men with low-risk PCa are at high risk of pathological upgrading and upstaging at RP. PSA and percentage positive cores are associated with upgrading and upstaging. These findings should be taken into consideration when discussing treatment options with these patients.

## Conflicts of interest

The authors declare that they have no conflicts of interest.

## Funding

There was no funding for this study.

## Ethical approval

Ethical approval was obtained from the Institutional Review Board, University of the West Indies, Faculty of Medical Sciences, Mona, Jamaica.

## Figures and Tables

**Table 1.** Clinico-pathological characteristics of patients with low-risk prostate cancer managed with radical prostatectomy at the University Hospital of the West Indies, 2000–2015.

Clinical characteristics	
Age, years (*N* = 138): mean (range)	59.5 ± 7.8 (37–75)
PSA, ng/mL (*N* = 122): median	6.7
Clinical T stage (*N* = 96): *n* (%)	
T1c	79 (82.3)
T2a	17 (17.7)
Positive cores (*N* = 24): mean (range)	2 (1–6)
Percentage positive cores (*N* = 117): *n* (%)	
<10%	71 (60.7)
10%–20%	21 (17.9)
>20%	25 (21.4)
Pathological characteristics	
Upgrading (*N* = 118): *n* (%)	57 (48.3)
Gleason 7	56
Gleason 8	1
Upstaging (*N* = 140): *n* (%)	16 (11.4)
pT3aN0	1
pT3bN0	8
pN1	7
Positive margins (*N* = 132): *n* (%)	31 (23.5)
Extra-prostatic extension (*N* = 137): *n* (%)	4 (2.9%)
Seminal vesicle invasion (*N* = 140): *n* (%)	9 (6.4)
Lymph node invasion (*N* = 128): *n* (%)	7 (5.5)

**Table 2.** Bivariate analysis of predictors of pathological upgrading in men with low-risk prostate cancer treated with radical prostatectomy at the University Hospital of the West Indies, 2000–2015.

Characteristics	Mean ± SD/n (%)
Age (*p* = 0.0868)	
Upgraded	59.2 ± 7.8
Not upgraded	59.4 ± 8.3
PSA (*p* = 0.014)	
Upgraded	7.0
Not upgraded	6.0
Clinical stage (*p* = 0.912)	
cT1	29 (39.7)
cT2a	7 (41.2)
Percentage positive cores (*p* = 0.002)	
<10%	18 (32.7)
10%–20%	9 (47.5)
>20%	18 (75)

**Table 3.** Bivariate analysis of predictors of pathological upstaging in Jamaican men with low-risk prostate cancer treated at the University Hospital of the West Indies, 2000–2015.

Characteristic	Mean ± SD/n (%)
Age (*p* = 0.348)	
Upstaged	57.8 ± 8.0
Not upstaged	59.7 ± 6.4
Clinical stage (*p* = 0.201)	
cT1	10 (12.7)
cT2a	0 (0.0)
PSA (*p* = 0.042)	
Upstaged	7.8
Not upstaged	6.4
Percentage positive cores (*p* = 0.003)	
<10%	2 (2.8)
10%–20%	2 (9.5)
>20%	6 (25)

## References

[1] Gibson TN, Hanchard B, Waugh N (2010). Age-specific incidence of cancer in Kingston and St. Andrew, Jamaica, 2003–2007. West Indian Med J.

[2] Powell IJ, Bollig-Fischer A (2013). Minireview: the molecular and genomic basis for prostate cancer health disparities. Mol Endocrinol.

[3] Blake G, Hanchard B, Mitchell K (2002). Jamaica cancer mortality statistics, 1999. West Indian Med J.

[4] Brawley OW (2012). Trends in prostate cancer in the United States. J Natl Cancer Inst Monogr.

[5] Coard KC, Skeete DH (2009). A 6-year analysis of the clinicopathological profile of patients with prostate cancer at the University Hospital of the West Indies, Jamaica. BJU Int.

[6] Shirley SE, Escoffery CT, Sargeant LA (2002). Clinicopathological features of prostate cancer in Jamaican men. BJU Int.

[7] Glover FE, Coffey DS, Douglas LL (1998). The epidemiology of prostate cancer in Jamaica. J Urol.

[8] Morrison BF, Aiken WD, Reid ME (2011). Impact of the National Health Fund policy on hormone treatment for prostate cancer in Jamaica. Rev Panam Salud Publica.

[9] Maurice MJ, Sundi D, Schaeffer EM (2017). Risk of pathological upgrading and up staging among men with low risk prostate cancer varies by race: results from the national cancer database. J Urol.

[10] Dinh KT, Mahal BA, Ziehr DR (2015). Incidence and predictors of upgrading and up staging among 10,000 contemporary patients with low risk prostate cancer. J Urol.

[11] Sundi D, Ross AE, Humphreys EB (2013). African American men with very low-risk prostate cancer exhibit adverse oncologic outcomes after radical prostatectomy: should active surveillance still be an option for them?. J Clin Oncol.

[12] Ha YS, Salmasi A, Karellas M (2013). Increased incidence of pathologically nonorgan confined prostate cancer in African-American men eligible for active surveillance. Urology.

[13] Leapman MS, Freedland SJ, Aronson WJ (2016). Pathological and biochemical outcomes among African-American and Caucasian men with low risk prostate cancer in the SEARCH database: implications for active surveillance candidacy. J Urol.

[14] Mohler J, Bahnson RR, Boston B (2010). NCCN clinical practice guidelines in oncology: prostate cancer. J Natl Compr Canc Netw.

[15] Meunier ME, Eyraud R, Senechal C (2017). Active surveillance for favorable risk prostate cancer in African Caribbean men: results of a prospective study. J Urol.

[16] Hwang I, Lim D, Jeong YB (2015). Upgrading and upstaging of low-risk prostate cancer among Korean patients: a multicenter study. Asian J Androl.

[17] Park JW, Koh DH, Jang WS (2018). Predictors of adverse pathologic features after radical prostatectomy in low-risk prostate cancer. BMC Cancer.

[18] de Cobelli O, Terracciano D, Tagliabue E (2015). Body mass index was associated with upstaging and upgrading in patients with low-risk prostate cancer who met the inclusion criteria for active surveillance. Urol Oncol.

[19] Jalloh M, Myers F, Cowan JE (2015). Racial variation in prostate cancer upgrading and upstaging among men with low-risk clinical characteristics. Eur Urol.

[20] Qi R, Moul J (2017). African American men with low-risk prostate cancer are candidates for active surveillance: the will-rogers effect?. Am J Mens Health.

[21] Dinizo M, Shih W, Kwon YS (2018). Multi-institution analysis of racial disparity among African-American men eligible for prostate cancer active surveillance. Oncotarget.

[22] Philip J, Ragavan N, Desouza J (2004). Effect of peripheral biopsies in maximising early prostate cancer detection in 8-, 10- or 12-core biopsy regimens. BJU Int.

[23] Sundi D, Kryvenko ON, Carter HB (2014). Pathological examination of radical prostatectomy specimens in men with very low risk disease at biopsy reveals distinct zonal distribution of cancer in black American men. J Urol.

[24] Tiguert R, Gheiler EL, Tefilli MV (1998). Racial differences and prognostic significance of tumor location in radical prostatectomy specimens. Prostate.

[25] Majoros A, Szasz AM, Nyirady P (2014). The influence of expertise of the surgical pathologist to undergrading, upgrading, and understaging of prostate cancer in patients undergoing subsequent radical prostatectomy. Int Urol Nephrol.

[26] Coard KC, Freeman VL (2004). Gleason grading of prostate cancer: level of concordance between pathologists at the University Hospital of the West Indies. Am J Clin Pathol.

[27] Aiken WD, Chin W (2015). Surgical access for radical retropubic prostatectomy in the phenotypically narrow and steep black male’s pelvis is exacerbated by a posterior pubic symphyseal protuberance: a case report. Int J Surg Case Rep.

[28] Powell IJ, Heilbrun LK, Sakr W (1997). The predictive value of race as a clinical prognostic factor among patients with clinically localized prostate cancer: a multivariate analysis of positive surgical margins. Urology.

[29] von Bodman C, Matikainen MP, Yunis LH (2010). Ethnic variation in pelvimetric measures and its impact on positive surgical margins at radical prostatectomy. Urology.

[30] Rabbani F, Yunis LH, Vora K (2009). Impact of ethnicity on surgical margins at radical prostatectomy. BJU Int.

[31] Sanda MG, Cadeddu JA, Kirkby E (2018). Clinically localized prostate cancer: AUA/ASTRO/SUO guideline. Part I: risk stratification, shared decision making, and care options. J Urol.

[32] Mahal BA, Aizer AA, Ziehr DR (2014). Racial disparities in prostate cancer-specific mortality in men with low-risk prostate cancer. Clin Genitourin Cancer.

[33] Iremashvili V, Soloway MS, Rosenberg DL (2012). Clinical and demographic characteristics associated with prostate cancer progression in patients on active surveillance. J Urol.

[34] Abern MR, Bassett MR, Tsivian M (2013). Race is associated with discontinuation of active surveillance of low-risk prostate cancer: results from the Duke Prostate Center. Prostate Cancer P D.

[35] Sundi D, Faisal FA, Trock BJ (2015). Reclassification rates are higher among African American men than Caucasians on active surveillance. Urology.

[36] Lozano R, Naghavi M, Foreman K (2012). Global and regional mortality from 235 causes of death for 20 age groups in 1990 and 2010: a systematic analysis for the Global Burden of Disease Study 2010. Lancet.

[37] Jemal A, Bray F, Center MM (2011). Global cancer statistics. CA Cancer J Clin.

[38] Pessoa RR, Viana PC, Mattedi RL (2017). Value of 3-Tesla multiparametric magnetic resonance imaging and targeted biopsy for improved risk stratification in patients considered for active surveillance. BJU Int.

[39] Kim HL, Li P, Huang HC (2018). Validation of the Decipher Test for predicting adverse pathology in candidates for prostate cancer active surveillance. Prostate Cancer P D.

[40] Mitsuzuka K, Narita S, Koie T (2013). Pathological and biochemical outcomes after radical prostatectomy in men with low-risk prostate cancer meeting the Prostate Cancer International: active surveillance criteria. BJU Int.

